# Across Kingdoms: The Bacteriome, Mycobiome, and Virome in Autoimmune Diseases: Mechanistic Insights, Therapeutic Perspectives, and the Emerging Role of COVID-19

**DOI:** 10.3390/nu18122032

**Published:** 2026-06-22

**Authors:** Edit Posta, Eva Gyarmati, Laszlo Majoros, Istvan Fekete, Istvan Varkonyi, Eva Zold, Zsolt Barta

**Affiliations:** 1 Department of Infectology, Faculty of Medicine, University of Debrecen, Bartok Bela u 2-26, 4031 Debrecen, Hungary; 2 Doctoral School of Clinical Immunology and Allergology, Faculty of Medicine, University of Debrecen, Nagyerdei krt. 98, 4032 Debrecen, Hungary; 3 Department of Microbiology, Faculty of Medicine, University of Debrecen, Nagyerdei krt. 98, 4032 Debrecen, Hungary; 4 Institute of Food Technology, Faculty of Agricultural and Food Sciences and Environmental Management, University of Debrecen, Boszormenyi ut 138, 4032 Debrecen, Hungary; 5 Department of Clinical Immunology, Institute of Internal Medicine, Faculty of Medicine, University of Debrecen, Moricz Zsigmond krt. 22, 4032 Debrecen, Hungary

**Keywords:** microbiome, autoimmunity, systemic autoimmune diseases, rheumatoid arthritis, systemic lupus erythematosus, COVID-19, diet, nutrition, probiotics, postbiotics

## Abstract

Autoimmune and immune-mediated inflammatory diseases (IMIDs) develop when genetically and environmentally susceptible hosts lose stable immune tolerance. The gut ecosystem is increasingly recognized as a biologically active interface in this process. Its bacterial, fungal, and viral components may shape mucosal and systemic immunity through antigenic stimulation, barrier regulation, and metabolite-dependent signaling, although the strength of evidence is uneven: bacteriome data are currently the most mature, whereas mycobiome, virome, and phageome findings remain more disease-specific and emerging. Dysbiosis may influence autoimmunity through overlapping routes, including epithelial barrier failure, altered short-chain fatty acid, bile acid, and tryptophan metabolism, molecular mimicry, and cross-kingdom microbial interactions. Nutrition is central to this network because dietary substrates determine microbial growth, metabolic output, epithelial integrity, and immune-cell differentiation. In this narrative review, we integrate evidence on disease-associated bacteriome, mycobiome, and virome patterns in systemic autoimmune diseases, with emphasis on rheumatoid arthritis, systemic lupus erythematosus, Sjögren’s syndrome, systemic sclerosis, spondyloarthritis, vasculitides, and idiopathic inflammatory myopathies. COVID-19 is considered not as a proven causal driver of autoimmunity, but as an example of an environmental and infectious insult capable of perturbing microbiome–barrier–immune communication. Finally, we discuss diet-based and microbiome-targeted approaches, including probiotics, prebiotics, synbiotics, and postbiotics, as adjunctive strategies that may help restore microbial resilience and immune balance. A better understanding of the diet–microbiome–host immunity axis may support more personalized preventive and therapeutic concepts in autoimmune disease.

## 1. Introduction

Autoimmune diseases and immune-mediated inflammatory diseases (ADs/IMIDs) comprise a clinically diverse group of chronic inflammatory disorders, including rheumatoid arthritis (RA), systemic lupus erythematosus (SLE), Sjögren’s syndrome (pSS), systemic sclerosis (SSc), polymyositis/dermatomyositis (PM/DM), mixed connective tissue disease (MCTD), and spondyloarthritis (SpA). Despite their different target organs and clinical phenotypes, these diseases share a common biological background: genetic predisposition interacts with infections, environmental exposures, lifestyle, mucosal immune activation, and disturbed tolerance mechanisms [1].

Within this framework, the human microbiome has become a key immunological regulator rather than a passive bystander. Bacterial, fungal, and viral communities participate in antigen presentation, lymphocyte polarization, effector-cell activity, and molecular mimicry [2]. Their metabolites, including short-chain fatty acids (SCFAs), secondary bile acids, and tryptophan-derived compounds, influence both innate and adaptive immunity. When microbial ecology is disturbed, barrier function and immune homeostasis may deteriorate, creating conditions that favor chronic inflammation and loss of self-tolerance [3].

**Figure 1:**
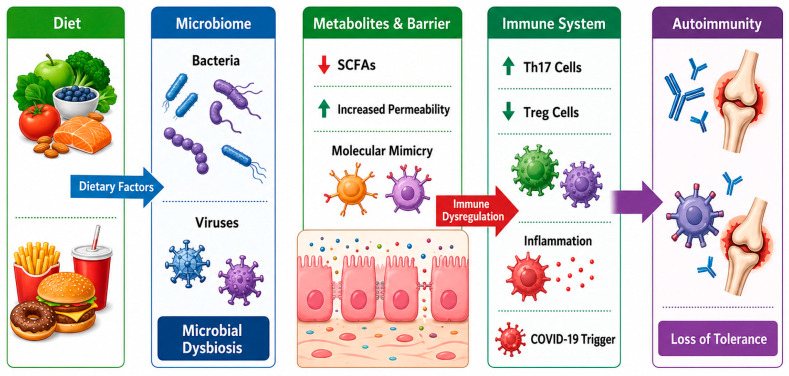
Conceptual framework of diet–microbiome–metabolite–barrier–immune interactions in autoimmune diseases. Diet shapes bacterial, fungal, and viral communities and thereby influences microbial metabolites, epithelial permeability, immune regulation, inflammation, and loss of tolerance. Arrows indicate the proposed direction of interaction between conceptual domains, and colors distinguish the diet, microbiome, metabolite/barrier, immune-system, and autoimmunity components.

This review summarizes current evidence connecting intestinal microbial communities, cross-kingdom microbial interactions, nutrition, and host immunity in ADs and IMIDs. We use the term “cross-kingdom” to denote an interacting ecological network in which bacteria, fungi, viruses, and bacteriophages may influence one another and the host, rather than to imply that all kingdoms are equally well studied or equally causal in every disease. Particular attention is given to COVID-19 as a potential microbiome-disrupting and immune-modulating event because SARS-CoV-2 infection can affect epithelial barriers, systemic inflammation, antiviral immune responses, and the composition of bacterial, fungal, and viral gut communities. Although the main focus is systemic autoimmunity, gastrointestinal immune-mediated conditions such as celiac disease and inflammatory bowel disease serve as useful mechanistic models. They illustrate how diet, microbial ecology, epithelial permeability, tolerance breakdown, mimicry, and metabolite signaling may converge in immune-mediated pathology as summarized in Figure 1.

The conceptual aim is therefore twofold: first, to distinguish well-supported bacteriome-associated pathways from more preliminary mycobiome and virome observations; and second, to identify where cross-kingdom interactions may provide plausible mechanistic links, biomarkers, or future therapeutic hypotheses. Throughout the review, observational associations are distinguished from experimental or interventional evidence, and disease-specific sections explicitly indicate when the available evidence remains exploratory.

## 2. Methods

This narrative review was prepared to integrate evidence on bacterial, fungal, and viral components of the gut microbiome in autoimmune and immune-mediated inflammatory diseases, with a specific emphasis on diet–microbiome–immune interactions and on the possible contribution of SARS-CoV-2/COVID-19 to this axis. PubMed/MEDLINE was searched from database inception to 31 March 2026. Representative search combinations included: (“gut microbiota” OR microbiome OR bacteriome OR mycobiome OR virome OR phageome) AND (autoimmunity OR “autoimmune disease” OR “immune-mediated inflammatory disease”); (“gut microbiota” OR microbiome) AND (rheumatoid arthritis OR systemic lupus erythematosus OR Sjögren OR systemic sclerosis OR spondyloarthritis OR myositis OR dermatomyositis OR polymyositis OR vasculitis); (diet OR nutrition OR fiber OR Mediterranean diet OR probiotic OR prebiotic OR synbiotic OR postbiotic OR “short-chain fatty acid”) AND (microbiome OR autoimmunity); and (COVID-19 OR SARS-CoV-2) AND (autoimmunity OR microbiome OR dysbiosis OR mycobiome OR virome).

Eligible publications included original human studies, relevant animal and translational studies, systematic reviews, meta-analyses, and high-quality narrative reviews when they provided clinically or mechanistically relevant information. We prioritized studies that linked microbial alterations to immune, metabolic, barrier-related, cross-kingdom, or nutritional pathways. Exclusion criteria were: articles not written in English, conference abstracts without sufficient methodological detail, studies unrelated to autoimmune or immune-mediated inflammatory disease, reports focused only on technical microbiome methodology without disease relevance, and purely taxonomic studies when no biological, clinical, or mechanistic interpretation was possible. Studies describing only taxonomic differences were included selectively when they addressed a poorly represented disease area or provided data relevant to bacteria–fungi, bacteria–phage, diet–microbiome, or microbiome–immune interactions.

Titles, abstracts, and full texts were screened for relevance to the conceptual scope of the review. Because the aim was integrative rather than systematic, formal risk-of-bias scoring and quantitative meta-analysis were not performed. The literature was not suitable for quantitative pooling because studies differed substantially in design, population, disease activity, medication exposure, dietary assessment, microbiome methodology, sample type, sequencing strategy, and outcome reporting. Findings were therefore synthesized narratively, with the aim of identifying recurring microbial signatures, disease-specific patterns, shared immunopathogenic mechanisms, interkingdom interactions, and possible diet- or microbiome-based therapeutic directions.

To reduce overinterpretation, evidence was interpreted according to its level of support. Experimental intervention studies, longitudinal human studies, mechanistic animal models, and in vitro studies were considered stronger indicators of biological plausibility, whereas cross-sectional microbiome associations were interpreted as hypothesis-generating. Throughout the manuscript, microbial findings are therefore discussed as potential triggers, amplifiers, biomarkers, therapeutic modifiers, or consequences of disease according to the available evidence. As with all narrative reviews, selection bias cannot be excluded, but the revised synthesis aims to make the selection process and evidentiary limitations more explicit.

## 3. Gut Permeability, Dysbiosis, and Cross-Kingdom Microbial Interactions

Dysbiosis, broadly defined as an imbalance in microbial community structure or function, has been repeatedly linked to ADs and IMIDs. A meta-analysis including 92 studies did not identify one universal autoimmune microbial signature. Instead, it pointed to recurring patterns across conditions: depletion of beneficial commensals, enrichment of inflammatory pathobionts, impaired barrier function, and altered microbial metabolite profiles [4]. These changes may affect more than epithelial permeability. Microbial communities also influence IgA production through intestinal dendritic cells [5], GLP-1 secretion, and GLP-1 receptor signaling [6,7]. Increased intestinal permeability, often described as “leaky gut”, allows microbial products, food antigens, toxins, and cell-wall components to move across the epithelial barrier [8]. Zonulin is involved in tight-junction regulation, and elevated serum or fecal zonulin correlates with permeability and bacterial translocation [9]. Experimental work suggests that apple-derived pectin may strengthen the barrier and reduce endotoxemia-related metabolomic signals [10]. Clinical synthesis also indicates that probiotics and synbiotics can reduce serum zonulin and may improve permeability in some settings [11]. Once microbial components such as LPS or flagellin cross the barrier, they can activate pattern-recognition receptors, particularly TLR4 and TLR5, with downstream NF-κB-driven cytokine production [12]. Dysbiosis and infection may further promote epithelial apoptosis, TNF-α release, and Th17/Treg imbalance [13]. Bacterial extracellular vesicles provide another route by which proteins, enzymes, lipids, toxins, virulence factors, and nucleic acids can reach host immune pathways and behave as pathogen-associated molecular patterns [14,15].

The autoimmune relevance of the microbiome is not confined to bacteria. Fungal and viral communities may also influence immune activation. Dysbiosis can reshape both the mycobiome and virome, and a *Candida albicans*-dominated enterotype has been associated with aging, impaired gut permeability, and higher disease risk [16]. *C. albicans* may induce autoreactive Th17 responses that cross-react with other fungi; this has been proposed as a link between *C. albicans* immunity and *Aspergillus fumigatus*-associated allergic bronchopulmonary aspergillosis [17]. Alcohol-related barrier injury provides another example of fungal involvement: alcoholic hepatitis has been associated with reduced fungal diversity, *Candida* enrichment relative to Penicillium-dominated communities, and increased anti-*Saccharomyces cerevisiae* antibody titers [18].

The gut virome is composed mainly of bacteriophages, with smaller contributions from eukaryotic viruses and plant-derived dietary or environmental viruses. Phage diversity changes during childhood and usually stabilizes in adulthood [19]. Disease-related virome patterns are increasingly being described [20]. In a Japanese case–control study, crAss-like phages, which are considered part of a healthy gut ecosystem, were reduced in both RA and SLE, while Podoviridae members were depleted in SLE [21]. SARS-CoV-2 infection adds another layer of disruption. COVID-19 has been linked to unstable fungal community structure and persistent mycobiome dysbiosis in some patients [22]. More broadly, COVID-19-associated dysbiosis involves reduced diversity, loss of beneficial commensals, expansion of opportunistic organisms, increased permeability, systemic inflammation, and immune dysregulation. These alterations may extend beyond the bacteriome to the virome and phageome [19,23].

A cross-kingdom interpretation is most useful when these microbial compartments are considered as an interacting network rather than as parallel lists of organisms. Bacteria generate metabolites such as SCFAs, bile acid derivatives, and tryptophan metabolites that regulate barrier and immune function. Fungi provide cell-wall ligands such as β-glucans and mannans can modify Th17-related mucosal immunity and may expand when bacterial communities are disturbed. Bacteriophages can reshape bacterial population structure, horizontal gene transfer, and microbial metabolic capacity, whereas eukaryotic viruses may injure epithelial barriers or alter interferon-dominated immune tone. Thus, cross-kingdom dysbiosis may act through at least four converging routes: altered metabolite output, increased epithelial permeability, antigenic mimicry or bystander activation, and phage- or virus-mediated remodeling of bacterial ecology. This model is mechanistically plausible, but its empirical support varies substantially among diseases.

Dietary substrates are among the most direct modulators of microbial metabolism, epithelial integrity, and immune tone. This explains the growing interest in nutritional and microbiome-targeted interventions, including probiotics, prebiotics, and postbiotics. Nutrient availability shapes which microbial taxa expand, and which bioactive metabolites are generated. Fiber intake, for example, supports SCFA-producing organisms such as *Faecalibacterium prausnitzii* and members of the Lachnospiraceae family. Butyrate and other SCFAs promote regulatory T-cell differentiation, reinforce epithelial barrier function, and counterbalance pro-inflammatory Th17 responses [24,25,26]. SCFAs may also influence TLR signaling, neutrophil activity, and regulatory T-cell biology, making this pathway relevant to autoimmune progression [27]. Diet also modulates microbial bile acid and tryptophan metabolism, producing ligands that act through nuclear receptors and aryl hydrocarbon receptor (AhR) signaling [28]. In contrast, Western-type diets rich in refined carbohydrates, ultra-processed foods, and saturated fat may favor dysbiosis, inflammation, insulin resistance, dyslipidemia, and renin–angiotensin system activation. Experimental and clinical observations support a pro-inflammatory role for such dietary patterns in gut microbial ecology [29].

Taken together, autoimmune diseases appear to share a set of microbiome-associated mechanisms rather than one disease-defining microbial pattern. These mechanisms include loss of SCFA-producing commensals, pathobiont expansion, increased permeability, altered metabolite output, Th17/Treg imbalance, molecular mimicry, and bacteria–fungi or bacteria–phage interactions. The framework summarized in Table 1 and Table 2 therefore presents autoimmunity as a group of clinically distinct but biologically overlapping disorders in which microbial ecology may help shape immune dysregulation.

The following sections review disease-specific microbial patterns and summarize possible points of nutritional or microbiome-targeted modulation.

### 3.1. Rheumatoid Arthritis

RA is a systemic immune-mediated disease characterized by chronic destructive synovitis, most often involving the small joints of the hands and feet. Extra-articular manifestations may include Felty syndrome, vasculitis, scleritis, secondary Sjögren’s syndrome, neuropathy, inflammatory lung disease, glomerulonephritis, osteoporosis, and increased cardiovascular risk [75,76]. Classification relies on the 2010 EULAR/ACR criteria [77]. Seropositive disease is defined by rheumatoid factor and/or anti-citrullinated protein antibodies (ACPA), whereas seronegative RA is clinically more heterogeneous and may involve different immunological pathways [78]. RA develops through a combination of genetic susceptibility, infections, environmental exposures, and mucosal immune activation [79].

Among gut microbes, *Prevotella copri* has attracted particular attention. RA patients may show IgG and IgA responses against *P. copri* antigens, and *P. copri* DNA has been detected in synovial fluid [30]. Anti-Pc-p27 IgA and IgG responses were reported at higher levels in individuals positive for both ACPA and RF, supporting a possible role in preclinical synovitis [31]. Stool sequencing has identified at least two RA-associated enterotype patterns: a *Prevotella*-dominant RA E1 type and a *Bacteroides*-dominant RA E2 type. Although inflammatory markers and cytokines did not differ substantially between these groups, CD8+ T-cell counts were higher in RA E2, and RA E1 patients appeared to respond more favorably to methotrexate (MTX) [32]. Additional data suggest that *Bacteroides fragilis* may influence MTX efficacy. MTX-associated reduction in B. fragilis was linked to lower therapeutic effect, while B. fragilis transplantation restored efficacy in collagen-induced arthritis. A proposed mechanism involves butyrate production, M2 macrophage polarization, and PI3K/Akt-mediated anti-inflammatory signaling [33]. More generally, SCFAs help maintain Th17/Treg equilibrium through GPR41 and GPR43 activation and induction of IL-10 and TGF-β, providing a rationale for SCFA supplementation or personalized prebiotic/probiotic strategies in RA remission maintenance [35].

The oral–gut axis is also relevant to RA. Periodontal disease is an established environmental risk context, and *Porphyromonas gingivalis* is one of its best-studied pathobionts [80]. *P. gingivalis* can damage gingival epithelium, impair gut barrier function, and has been associated with arthritis flares [81,82]. B cells located in gingival mucosa may generate antibodies against citrullinated bacterial epitopes, thereby promoting ACPA production through molecular mimicry; NETosis may amplify this process [36,37]. Bacterial extracellular vesicles from *P. gingivalis*, and possibly from *Fusobacterium nucleatum* and *Aggregatibacter actinomycetemcomitans*, can further promote barrier injury and inflammatory cytokine release [15].

RA-associated fungal and viral communities remain less well defined, but available studies suggest potential relevance. In one Chinese cohort, *Candida* tended to be more frequent in RA, while *Pholiota*, *Scedosporium*, and *Trichosporon* were reduced; overall fungal diversity did not differ significantly, and the pathogenic significance remains uncertain [83]. Oral and gut virome analyses have also shown differences between RA patients, treated RA patients, and controls, including reduced oral viral richness after treatment and disrupted virus–bacterium interaction networks [84]. Bacteriophage composition may distinguish RA from controls and may relate to ACPA positivity and HLA-DR shared epitope status. *Prevotella*- and Oscillibacter-related phages have been proposed to stimulate CD4+ T-cell responses, while Lachnospiraceae- and Bacteroidaceae-targeting phages may have biomarker potential [85,86].

COVID-19 may intersect with RA through several mechanisms, including post-infectious immune activation, molecular mimicry involving spike protein and synovial antigens, and infection-associated gut dysbiosis [87,88]. However, not all epidemiological data support a direct association: one prospective cohort did not find SARS-CoV-2 infection to be associated with RA-related seropositivity or classifiable RA outcomes [89]. Therefore, the relationship between COVID-19, dysbiosis, and RA onset requires careful longitudinal evaluation. Nutritionally, high-fat, low-fiber Western dietary patterns may promote permeability and Th17-skewed inflammation, whereas fiber-rich and Mediterranean-style patterns may support SCFA-producing taxa and tolerogenic signaling. Probiotics, prebiotics, synbiotics, and postbiotics, especially butyrate-based approaches, may become adjunctive tools alongside standard RA therapy, but the clinical evidence remains heterogeneous.

In RA, the current evidence should therefore be interpreted along a continuum. Periodontal disease, *P. gingivalis*-related citrullination, and selected animal or transplantation studies provide stronger mechanistic plausibility, whereas many fecal microbiome signatures remain associative and may reflect disease activity, treatment exposure, diet, or inflammation. *P. copri* and related phage signals are best viewed as candidate mucosal biomarkers and possible immune amplifiers until longitudinal and intervention studies clarify their temporal and causal role.

### 3.2. Spondyloarthritis

Spondyloarthritis (SpA) includes related disorders with shared genetic and clinical features, affecting the spine, sacroiliac joints, peripheral joints, entheses, and sometimes extra-articular tissues. Axial SpA is classified using ASAS criteria, which combine inflammatory back pain beginning before age 45 with either imaging evidence of sacroiliitis plus one SpA feature or HLA-B27 positivity plus at least two SpA features [90]. Radiographic sacroiliitis represents a later stage captured by the modified New York criteria [91]. Peripheral SpA criteria focus on peripheral arthritis, enthesitis, or dactylitis without dominant axial involvement [92].

The link between SpA and inflammatory bowel disease suggests a shared gut–joint axis, although the precise role of dysbiosis remains unresolved [38]. Lavage-based sampling from lower gastrointestinal sites has shown reduced richness and Shannon diversity in SpA, with enrichment of pro-inflammatory taxa and overlap with IBD-like patterns. Enterobacteriaceae were increased in both fecal and lower-GI samples, whereas *Succinivibrio* was more prominent in feces [39]. In a study using the GA-map Dysbiosis Test, dysbiosis was found in 87% of ankylosing spondylitis patients and was characterized by higher Proteobacteria, Enterobacteriaceae, Bacilli, *Streptococcus*, and Actinobacteria, with lower *Bacteroides* and Lachnospiraceae. These changes correlated with fecal calprotectin but not with HLA-B27, disease activity, or medication exposure [40]. *Ruminococcus gnavus*, an anaerobe within Lachnospiraceae, has also been associated with subclinical intestinal inflammation in SpA [41].

Mycobiome and virome alterations add complexity to the SpA microbiome. Because psoriasis and IBD often coexist with SpA, their fungal signatures may also be relevant [42,43]. In stool samples from ankylosing spondylitis patients, ITS2 sequencing showed higher Ascomycota, especially Dothideomycetes, and lower Basidiomycota driven mainly by reduced Agaricales, although the cohort was small [44]. Metagenomic virome analysis reported increased viral richness in AS, with enrichment of Gratiaviridae and Quimbyviridae and depletion of Drexlerviridae and Schitoviridae. Since some enriched phages infect *Bacteroides*, phage–bacterial interactions may contribute to pathogenesis [45].

Several reactive arthritis, peripheral SpA, and axial SpA cases have been described after SARS-CoV-2 infection, but the mechanism remains uncertain and HLA-B27 status has not been consistently documented [93,94]. COVID-19-related intestinal dysbiosis is therefore a plausible, but unproven, contributor. From a nutritional standpoint, SpA is particularly relevant because subclinical gut inflammation and barrier dysfunction are common. Diets low in fermentable fiber and high in processed food may favor inflammatory microbial communities, whereas plant-rich patterns may increase microbial diversity and SCFA production. Prebiotics, fiber-based interventions, and selected probiotics could theoretically support epithelial integrity and mucosal immune regulation, especially in patients with gut symptoms, although robust SpA-specific intervention trials are still limited.

### 3.3. Systemic Lupus Erythematosus

SLE is a systemic autoimmune disease with marked clinical and immunological heterogeneity. The 2019 EULAR/ACR classification system requires a positive ANA as the entry criterion, followed by weighted clinical and immunological domains [95]. Experimental models suggest that gut dysbiosis may be more important for disease amplification than for disease initiation; for example, lactobacilli improved disease manifestations in female lupus-prone MRL/lpr mice when administered before disease onset [96].

Human SLE studies frequently link disease activity with reduced microbial diversity and dysbiosis. *Ruminococcus gnavus* has been reported at approximately fivefold higher abundance in SLE, and anti-*R. gnavus* antibody levels correlate with anti-dsDNA antibodies, lupus nephritis activity, and lower complement C3/C4 levels [46,47]. Other sequencing studies have described subgroup-specific bacterial patterns related to disease activity and treatment response. Although alpha diversity may be similar to controls, beta diversity and genus-level composition can differ, with increased *Muribaculaceae*, *Enterococcus*, *Akkermansia*, *Asteroleplasma*, *Clostridia UCG-014*, *Dialister*, *Holdemanella*, and *Parasutterella UCG-002*, several of which may have pro-inflammatory potential [48].

Metabolic immune dysregulation appears closely connected to the SLE microbiome. Pathobionts such as *L. reuteri*, *R. gnavus*, and *Enterococcus gallinarum* may expand in SLE or lupus models and translocate to lymph nodes, blood, liver, or spleen, where they can promote autoantibody production, TLR7 signaling, and altered immunometabolism. A lower *Firmicutes*/*Bacteroidetes* ratio has been linked to increased oxidative phosphorylation and glycan utilization, suggesting that microbial metabolites may influence immune-cell metabolism and barrier integrity [49]. Mycobiome data also point to altered fungal ecology. In fecal metagenomic sequencing, *Candida*, *Malassezia*, and *Trichophyton* were enriched, *Pichia* was depleted, and fungal diversity increased. Differences in fungal biosynthetic products, including kynurenine, secondary bile acids, terpenes, and post-translationally modified peptides, may affect bacterial–fungal and host–microbe interactions [50]. Similar fungal shifts, including increased *Candida* and altered Basidiomycota/Ascomycota ratios, have been reported in lupus mouse models and correlated with disease activity [97].

The SLE virome may also participate in immune activation. Fecal virus-like particles from untreated SLE patients were compositionally different mainly at the bacteriophage level, with reduced crAss-like viruses. These particles induced stronger IFN-α responses in epithelial and primary immune cells, particularly neutrophils, T cells, and B cells, and the IFN-α-inducing capacity decreased after treatment [98]. COVID-19 may provide a trigger for lupus-like autoimmunity through B-cell activation, interferon production, and autoantibody generation, including increased ANA positivity after severe SARS-CoV-2 infection [99,100]. The specific contribution of COVID-19-associated dysbiosis to lupus onset remains to be clarified.

Dietary modulation in SLE is attractive because microbial metabolism, barrier function, and immunometabolism intersect in this disease. Fiber-, polyphenol-, and unsaturated-fatty-acid-rich diets may support tolerogenic microbial communities, whereas Western dietary patterns may aggravate endotoxemia and dysbiosis. SCFAs, tryptophan derivatives, and secondary bile acids are particularly relevant because they link diet, microbial activity, and immune-cell metabolism. Probiotics and prebiotics have shown anti-inflammatory and barrier-protective effects in experimental lupus models, but human evidence remains limited. Postbiotics may be especially appealing in SLE, where defined microbial products or metabolites could offer immunomodulation without the uncertainties of live organisms.

In SLE, the distinction between trigger, amplifier, biomarker, and consequence is particularly important. *R. gnavus* expansion and anti-*R. gnavus* antibodies correlate with immunological activity and lupus nephritis in some cohorts, but these data do not prove that this taxon initiates disease. Similarly, viral or fungal signatures may reflect an interferon-rich inflammatory environment as much as they contribute to it. Longitudinal sampling before flare, after treatment, and during remission will be required to define directionality.

### 3.4. Sjögren’s Syndrome

Primary Sjögren’s syndrome (pSS) is a systemic autoimmune disease characterized by exocrine gland dysfunction and sicca symptoms, classified by the 2016 EULAR/ACR criteria [101]. Extra-glandular involvement can include vasculitis, interstitial lung disease, renal involvement, and arthritis. Gastrointestinal manifestations such as esophageal dysmotility, gastroparesis, and pancreatic insufficiency are also encountered, and pSS is strongly associated with celiac disease; screening for celiac disease is therefore clinically relevant [102].

Gut dysbiosis has been linked to pSS activity. In a stool 16S rRNA study of 42 pSS patients and 35 controls, severe dysbiosis was associated with higher ESSDAI and ClinESSDAI scores, lower C4, and elevated fecal calprotectin [51]. A comparison of pSS, primary biliary cholangitis, and healthy controls found reduced alpha diversity in both autoimmune groups but with disease-specific microbial patterns. *Ruminococcus torques*, *Clostridium celatum*, and *Lactobacillus vaginalis* correlated positively with liver enzymes in PBC, whereas *Faecalibacterium prausnitzii* correlated negatively with GGT and ALT in pSS [52]. These findings support the idea that microbiome-targeted intervention may eventually be disease-tailored rather than generic.

The oral microbiome is particularly important in pSS. Salivary dysbiosis may alter the antigen-presenting behavior of salivary gland epithelial cells. *Haemophilus parainfluenzae* is reduced in pSS, and in vitro it increased PD-L1 expression on A253 cells, suggesting a possible immunoregulatory role [53]. Salivary microbiome disruption can also occur in non-pSS hyposalivation, indicating that dryness itself may confound disease-specific patterns [54]. *Candida albicans* infection has been associated with increased pSS risk in a large Taiwanese cohort. Mechanistically, candidalysin can damage oral epithelium and stimulate Th17 activation, while galectin-3, which contributes to C. albicans phagocytosis, is elevated in pSS serum [55]. Virome data add another possible mechanism: *Vientovirus* abundance has been associated positively with anti-Ro52 and negatively with Schirmer test values, and its capsid peptides resemble SSA/Ro52 B-cell epitopes [56].

SARS-CoV-2 infection or vaccination has been described in temporal association with pSS or Sjögren-like disease in case reports and experimental work [103,104]. In ACE2-transgenic mice, infection reduced salivation and produced lymphocytic infiltration of lacrimal and salivary glands, together with increased ANA and SS-B/La positivity. In human sera, ANA and SSA/SSB/Ro52 positivity were increased after COVID-19 [105]. Spike-protein treatment in mice also promoted B-cell and effector-memory CD4+ T-cell infiltration of submandibular glands, STAT3 phosphorylation, and ANA positivity [106].

Because depression and anxiety are common in pSS, the gut–brain axis may be clinically relevant. Microbial metabolites can influence enteric, autonomic, neuroendocrine, neuroimmune, and behavioral pathways [107]. Nutritionally, pSS represents both an intestinal and oral microbiome disorder. Fiber-rich anti-inflammatory diets may support barrier-protective metabolites, whereas ultra-processed foods may worsen low-grade inflammation. Probiotics and synbiotics may be useful as supportive strategies for gut permeability and mucosal immune modulation, but well-controlled studies are still needed to determine whether these interventions can alter disease activity or extra-glandular features.

### 3.5. Systemic Sclerosis

Systemic sclerosis (SSc) is a rare autoimmune disease characterized by microvascular injury, endothelial dysfunction, immune activation, myofibroblast activation, and fibrosis. Gastrointestinal involvement is extremely common and may include esophageal dysfunction, anorectal involvement, small-bowel dysmotility, malabsorption, SIBO, pain, and diarrhea [57,63]. Because antigen-presenting cells and mucosal immune responses are influenced by microbial ecology, dysbiosis may contribute to both gastrointestinal burden and systemic disease behavior.

Several studies have identified altered gut bacterial communities in SSc. Patients with gastrointestinal manifestations show increased *Lactobacillus*, *Blautia*, and *Coprococcus* and lower *Roseburia* and *Faecalibacterium* compared with controls; *L. reuteri* enrichment and reduced *Roseburia faecis* and *F. prausnitzii* have also been reported [57,58]. *Prevotella copri* has been associated with malnutrition and fecal incontinence, whereas *Akkermansia muciniphila* has been linked to severe diarrhea in a 16S rRNA study of SSc patients and controls [59]. In SSc with SIBO, alpha diversity differed from controls, with higher *Bacteroides* and unclassified Rikenellaceae and lower unclassified Erysipelotrichaceae; anticentromere antibodies were not associated with SIBO or gastrointestinal involvement [60].

Dysbiosis appears early in the disease process. In very early diagnosis of SSc (VEDOSS), stool sequencing showed changes in *Bacteroidales*, *Oscillospirales*, *Bacilli*, *Blautia*, *Romboutsia*, *Streptococcus*, and *Turicibacter*, while established SSc showed enrichment of Acidaminococcaceae and *Sutterellaceae* and depletion of *Peptostreptococcaceae*, *Anaerostipes*, *Blautia*, *Romboutsia*, and *Turicibacter*. Both VEDOSS and SSc were associated with reduced butyrate and increased acetate [61]. Two independent cohorts from UCLA and Lund also demonstrated altered microbial composition, reduced *Faecalibacterium*, increased *Desulfovibrio* and *Ruminococcus*, and associations with immunosuppressant use, SIBO, and interstitial lung disease [62].

The SSc mycobiome and virome remain insufficiently studied, and no robust disease-defining fungal or viral signature has yet been established. COVID-19 has been temporally associated with scleroderma-like disease after infection and vaccination, with proposed mechanisms including endothelial injury, cytokine activation, molecular mimicry, and immune dysregulation [108,109,110]. The vascular similarities between COVID-19 endothelial injury and early SSc make this area interesting, but current evidence remains mainly associative. Nutritionally, SSc requires a personalized approach because dysmotility, malabsorption, and SIBO may limit tolerance of standard high-fiber recommendations. When tolerated, fiber may support SCFA-producing commensals, but symptom-adapted strategies are often necessary. Probiotics, prebiotics, and postbiotics may have future value for dysbiosis, barrier dysfunction, and inflammation, but evidence remains limited and should be tested against clinically meaningful gastrointestinal and fibrotic outcomes.

### 3.6. Polymyositis and Dermatomyositis

Polymyositis and dermatomyositis are classified among idiopathic inflammatory myopathies (IIMs), a group of systemic autoimmune disorders involving skeletal muscle and, in many patients, skin, joints, and lung. Classic Bohan and Peter criteria were published in 1975, while updated EULAR/ACR criteria appeared in 2017 [111]. As in other rheumatic diseases, the gut microbiome is being investigated as a potential modifier of immune activity in PM/DM.

Available bacterial data suggest dysbiosis but remain early. Low-dose IL-2 studies reported lower microbial diversity in IIM compared with controls, with increased Lachnospiraceae and Pseudobutyrivibrio after IL-2 treatment but no major change in richness or overall diversity [64]. A Mendelian randomization analysis linked *Alloprevotella*, *Ruminococcaceae*, *Anaerotruncus*, *Sutterella*, and *Dialister* to PM/DM, and *Ruminococcaceae* correlated positively with disease activity, suggesting that selected *Ruminococcaceae* strains may be mechanistically relevant [65]. In inclusion body myositis, *Bacteroides* was enriched and proposed as an age-related biomarker, with additional changes involving *Clostridium* CAG 352 and *Eggerthella*, although alpha and beta diversity were not clearly different [66].

Dermatomyositis cohorts show additional signals. In adult DM, alpha diversity was reduced, the Bacteroidetes/Firmicutes balance shifted, and *Streptococcus*, *Lachnoclostridium*, and *Tyzzerella* were enriched. Patients with ILD-associated DM showed even lower microbial diversity, and Proteobacteria were enriched in the ILD/myositis-associated autoantibody subgroup, consistent with a dysbiotic barrier-disrupting pattern [67]. In juvenile DM, oral and fecal microbiomes differed from unaffected family members, with increased *Faecalibacterium* and *Ruminococcaceae*, enrichment of *Roseburia* and *Muribaculaceae*, and depletion of several *Streptococcus* sequences. The latter observation is notable because antibodies against *S. pyogenes* may cross-react with muscle-specific myosin M5 [68,69].

A whole-metagenome shotgun study of IIM provided cross-kingdom data. Although bacterial diversity did not differ significantly, community composition did. *Streptococcus parasanguinis* and *Akkermansia muciniphila* were enriched, whereas *Faecalibacterium prausnitzii*, *Megamonas funiformis*, and *Eubacterium rectale* were reduced. The fungal community also differed, with enrichment of opportunistic taxa such as *Aspergillus* sp. *c56*, *Trichophyton rubrum c61*, and *Candida boidinii c94*. Virome changes included increased Salasmaviridae, Metaviridae, and Siphoviridae and alterations in phage-related functions such as metabolic regulation and chromosomal maintenance [70].

Thus, the PM/DM and broader IIM literature should be regarded as exploratory. The available studies are small, heterogeneous, and often cross-sectional, and the cross-kingdom signal is stronger as a source of hypotheses than as evidence of causality. At present, bacterial, fungal, and viral findings in IIM are best interpreted as candidate biomarkers or modifiers of immune-metabolic state rather than established disease drivers.

COVID-19 intersects with IIM clinically because myalgia is common, while viral myositis, rhabdomyolysis, and dermatomyositis-like presentations are rare but described. Proposed mechanisms include ACE2-mediated muscle entry, innate and adaptive immune activation, autoinflammation, and cross-reactivity; anti-Mi2, anti-MDA5, and anti-SAE antibodies have been reported in some cases [112]. A Spanish multicenter observational study also described IIM, myositis-specific antibodies, and myositis-associated antibodies after infection or vaccination [113]. Diet–microbiome data in PM/DM are sparse, but anti-inflammatory dietary patterns rich in fiber, omega-3 fatty acids, and polyphenols may theoretically promote regulatory metabolites and epithelial integrity. Probiotics and prebiotics remain exploratory, while postbiotics may eventually provide a more targeted approach to immunometabolic modulation without the variability of live microbial products.

### 3.7. Systemic Vasculitides

Systemic vasculitides are immune-mediated disorders defined by inflammation of small, medium, large, or variable-size vessels [114]. Microbiome research is less mature in vasculitis than in RA or SLE, yet signals have emerged in Behçet’s disease, IgA vasculitis, Kawasaki disease, and ANCA-associated vasculitis (AAV). Across these conditions, the recurring pattern is loss of beneficial commensals, expansion of potentially inflammatory taxa, altered SCFA-producing communities, and activation of mucosal and systemic immune pathways [73]. Behçet’s disease is the most developed model. Gut dysbiosis in Behçet’s disease has been linked to reduced butyrate-producing bacteria and altered tryptophan metabolism, which may influence T-cell differentiation, epithelial barrier integrity, IL-22-related mucosal responses, and systemic vascular inflammation [72].

In AAV, microbiome work has focused primarily on upper and lower airway mucosal sites [115], but intestinal dysbiosis has also been described. In a small cross-sectional study, active AAV showed altered fecal abundance of *Dialister*, *Prevotella*, *Faecalibacterium*, and *Sutterella* compared with remission, while remission samples resembled healthy controls [116]. Additional work in AAV with kidney involvement found reduced alpha diversity and depletion of SCFA-producing taxa, including *Subdoligranulum*, *Eubacterium hallii*, *Ruminococcaceae UCG013*, *Eubacterium ventriosum*, *Dorea*, and *Butyricicoccus* [117].

IgA vasculitis, particularly in pediatric cohorts, has also been associated with disturbed intestinal microbial composition and inflammatory cytokine imbalance, supporting a possible link between gut dysbiosis, mucosal immune activation, and abnormal IgA responses [74]. Kawasaki disease offers a second gut–vascular example. Altered gut microbial profiles, reduced potentially protective SCFA-linked taxa, and enrichment of opportunistic pathogens have been reported, but it remains uncertain whether microbiome modification can influence vascular outcomes [71].

Large-vessel vasculitides are beginning to enter this field. Giant cell arteritis (GCA), the most common systemic vasculitis in Western populations, can cause segmental stenosis, occlusion, aneurysmal dilation, visual loss, TIA, or stroke. Mendelian randomization studies have suggested specific associations between gut microbiota and GCA [118,119]. Polymyalgia rheumatica, which is closely associated with GCA, has not yet been adequately explored from a microbiome perspective. Takayasu arteritis (TAK), another large-vessel vasculitis affecting the aorta and major branches, has been investigated in an integrated multi-omics study of 172 participants. *Escherichia* species and *Klebsiella pneumoniae* were enriched, *L. bacterium 7 1 58FAA* was associated with active disease, and some pathobionts were detected in inflamed aortic wall tissue. These observations raise the possibility of bacterial translocation or tissue-associated microbial biomarkers, but direct causal evidence is still lacking [120].

From a nutritional viewpoint, vasculitides fit naturally within the broader diet–microbiome–immune model. Fiber-rich, plant-forward diets may favor butyrate-producing bacteria and anti-inflammatory metabolites, whereas Western dietary patterns may reinforce dysbiosis and systemic inflammation. Probiotics, prebiotics, synbiotics, and postbiotics remain experimental in vasculitis, but they are plausible adjunctive strategies for future trials, especially in disorders with oral, gastrointestinal, mucosal, or gut–vascular involvement.

Overall, vasculitis represents one of the least mature areas of autoimmune microbiome research. Evidence for Behçet’s disease is stronger than for many other vasculitides, whereas data for AAV, IgA vasculitis, Kawasaki disease, GCA, polymyalgia rheumatica, and TAK remain fragmented. The current literature supports a gut–vascular hypothesis, but not yet a causal or interventional model. Future studies should separate mucosal biomarkers from true pathogenic mechanisms and should include longitudinal sampling, immune phenotyping, and dietary or metabolomic assessment.

### 3.8. Opportunities for Gut Microbiota Modulation in Autoimmune Diseases: Dietary Intervention and Modulation of the Microbiome–Immune Axis

Dietary intervention is one of the most clinically accessible ways to influence the gut microbiome–immune axis in autoimmune disease. The available evidence does not support a single universal “autoimmune diet”; rather, it suggests that dietary patterns may modify inflammatory tone, barrier integrity, microbial metabolite production, and possibly treatment response. For this reason, nutrition should be considered a biologically active component of microbiome-directed care, not merely a lifestyle background variable.

Among currently studied dietary models, the Mediterranean diet (MD) has the strongest translational rationale. Although UNESCO describes MD as a cultural set of food-related practices extending from landscape to table, its clinical pattern is characterized by frequent olive oil use, high intake of fruits, vegetables, legumes, nuts, and unrefined grains, moderate fish and poultry consumption, limited red meat and refined sugar, and low intake of ultra-processed foods. Its bioactive components include n-3 polyunsaturated fatty acids, monounsaturated fatty acids, polyphenols, and plant-derived fibers with prebiotic activity. These substrates may promote SCFA-producing organisms such as *Bifidobacterium*, *Roseburia*, and *Faecalibacterium prausnitzii*, improve epithelial barrier function, and reduce inflammatory signaling [121,122]. Extra-virgin olive oil, rich in oleic acid and other monounsaturated fatty acids, may additionally influence PPARα and PPARγ pathways, thereby linking diet to inflammation and metabolic regulation [122,123].

Omega-3 fatty acids represent another relevant nutritional strategy. Fish oil provides eicosapentaenoic acid and docosahexaenoic acid, which may reduce inflammatory lipid mediators and influence immune-cell function. Long-term fish-oil intake has been associated with lower RA risk in a prospective study and with reduced leukotriene B4 levels [124,125]. However, prevention data for MD and RA remain heterogeneous: one nine-year cohort found lower RA risk with higher MD adherence [126], whereas other studies did not confirm a clear protective association [127]. These mixed findings underline the need to distinguish disease prevention, disease activity modulation, and symptom improvement when evaluating nutritional interventions.

Clinical intervention data in RA are encouraging but still incomplete. In a randomized dietary trial, both MD and Irish healthy eating guidelines improved HAQ-DI and RAQoL, while physical activity improved more clearly in the MD arm [128]. In another trial involving 210 RA patients, MD improved body composition, DAS28-CRP, DAS28-ESR, and adiponectin compared with a regular diet [129]. These data suggest that MD may have value as an adjunctive approach, particularly when combined with standard pharmacological treatment and individualized lifestyle counseling.

Evidence is also emerging in systemic autoimmune diseases beyond RA. In SLE, higher MD adherence was associated with lower SLEDAI/SDAI scores and fewer cardiovascular risk factors in a cross-sectional cohort of 280 patients [130], although another study in women with SLE did not find clear associations with arterial stiffness or inflammatory markers [131]. An Italian SLE cohort similarly linked higher MD adherence with lower disease severity, but not with cardiovascular events [132]. In pSS, better MD adherence was associated with lower Ocular Surface Disease Index scores in a cohort of 75 patients, without a clear relationship with ESSPRI [133]. A second pSS study involving 114 patients reported that higher MD adherence and fatty-acid intake were associated with lower OSDI values [134], and another single-center study suggested that higher intake of MD-related components, particularly galactose and vitamins A and C, was associated with lower pSS risk [135]. These findings support the concept that dietary patterns may influence both systemic and mucosal manifestations of autoimmunity.

SSc and SpA provide useful examples of why dietary recommendations must be disease- and phenotype-specific. In a four-center Italian SSc cohort, poor MD adherence assessed by MEDAS was associated with more severe Raynaud phenomenon and digital ulcers, and MEDAS scores inversely correlated with depression and gastroesophageal reflux [136]. A Spanish SSc study also linked poor MEDAS-14 adherence with worse outcomes and reduced muscle mass [137]. In axial SpA, a six-month prospective study found that higher MD adherence, assessed by PREDIMED, was associated with lower ASDAS-CRP [138]. In these conditions, dietary intervention may act not only through systemic inflammation, but also through gastrointestinal symptoms, barrier function, body composition, and patient-reported outcomes.

More restrictive anti-inflammatory approaches have also attracted attention, but the evidence is less mature. The ITIS diet, a modified Mediterranean-style intervention designed to reduce the *n*-6/*n*-3 ratio, lower gluten exposure, avoid solanine-containing vegetables, and increase prebiotics, probiotics, ginger, turmeric, and black pepper, is being compared with MD in RA [139]. In a small trial of 44 RA patients, both ITIS and MD improved CDAI and VAS scores, but remission occurred only in the ITIS group [140]. The autoimmune protocol diet (AIP) is another popular strategy based on the hypothesis that food antigens may aggravate immune activation when intestinal permeability is increased. AIP usually involves elimination, reintroduction, and maintenance phases, with temporary exclusion of grains, legumes, nightshades, nuts, seeds, dairy products, eggs, coffee, alcohol, artificial additives, and NSAIDs. Although small studies have explored AIP in RA, Hashimoto thyroiditis, and IBD, the results remain inconsistent and methodologically limited [141]. Therefore, restrictive diets should be used cautiously, preferably with dietetic supervision, because unnecessary exclusion may increase nutritional risk and reduce adherence.

SSc illustrates the need for careful personalization. Standard high-fiber recommendations may be biologically attractive because fiber supports SCFA production, but they may not be tolerated in patients with severe dysmotility, bloating, SIBO, malabsorption, or fecal incontinence. Low-FODMAP strategies are effective in IBS-like conditions in some populations, yet SSc-specific data are limited. In a study of 66 SSc patients, more severe gastrointestinal symptoms were associated with dysbiosis and reduced alpha and beta diversity, but low-FODMAP adherence did not substantially change microbial composition, except for Enterococcus, and did not improve gastrointestinal symptoms [142]. This highlights an important principle: dietary intervention in autoimmune disease must be evaluated using clinically meaningful outcomes, including symptoms, nutritional status, inflammatory activity, microbiome function, and quality of life.

Microbiome-targeted nutritional strategies also include prebiotics, probiotics, synbiotics, and postbiotics. Prebiotic fibers may increase SCFA production and support barrier-protective commensals; probiotics and synbiotics may improve intestinal permeability in selected contexts [10,11]. Postbiotics are conceptually attractive because they deliver defined microbial metabolites, cell fragments, or bioactive compounds without administering live organisms. This may be relevant in autoimmune patients receiving corticosteroids, immunosuppressants, or biologic therapies. Nevertheless, efficacy, dosing, strain or compound selection, and disease-specific indications remain insufficiently established.

Safety must remain central. Probiotics are generally safe in healthy individuals, but caution is warranted in severely immunosuppressed patients, those with central venous catheters, severe mucosal barrier disruption, or critical illness. For future research, dietary and microbiome-targeted trials should move beyond broad dietary labels and include standardized dietary assessment, adherence monitoring, microbiome and metabolomic endpoints, medication stratification, and patient-centered outcomes. The most promising direction is not a universal diet for all autoimmune diseases, but a personalized nutritional framework that integrates disease phenotype, gastrointestinal involvement, baseline diet, medication exposure, microbial signatures, and metabolic readouts.

## 4. Discussion

This review emphasizes that the gut microbiome and host immunity interact across a broad spectrum of autoimmune diseases [2,3,4]. Individual conditions have their own microbial features, yet several recurrent patterns are visible across diseases: loss of beneficial commensals, pathobiont expansion, epithelial barrier dysfunction, and altered metabolite production [3,4,8,12,13]. These findings support a model in which dysbiosis may function as a biomarker, amplifier, consequence, or contributor to immune dysregulation depending on disease context and evidence type, rather than as a universal primary cause.

Reduced SCFA availability, disturbed bile acid metabolism, and abnormal metabolite-dependent signaling may weaken immune tolerance and sustain chronic inflammation [3,12,61]. At the same time, increased permeability permits microbial products to enter systemic immune circuits, where they can amplify innate and adaptive responses [8,9,12]. Molecular mimicry and bystander activation provide additional routes by which microbes may connect mucosal dysbiosis with systemic autoimmunity [13,62,63].

A central message of this synthesis is that autoimmune diseases should not be interpreted only as isolated organ-specific disorders. Many of them share microbiome-shaped immunological pathways, including Th17/Treg imbalance, barrier failure, and metabolite-driven immune modulation. These common pathways may help explain overlapping clinical manifestations and comorbidities among autoimmune conditions [3,4,8,12,61].

The bacterial microbiome is only one component of this system. Fungal and viral communities add further biological layers [16,17,18,19,20,21,22,23], although the depth of evidence is still uneven across diseases. A genuine cross-kingdom model requires attention to interactions: bacterial depletion may permit fungal expansion; fungal antigens may activate pattern-recognition receptors or Th17-dominant mucosal responses; bacteriophages may alter bacterial ecology, virulence, and metabolic capacity; and eukaryotic viral infections may perturb epithelial integrity and interferon signaling [17,19,20,21,22,23]. These interkingdom relationships make the host–microbiome interface more complex, but they also create new mechanistic hypotheses and potential biomarkers that require direct testing.

Diet is one of the most modifiable forces acting on this network. Food composition affects microbial structure, metabolic activity, epithelial integrity, and immune signaling [10,11,12]. Fiber-rich diets tend to support SCFA-producing organisms and tolerogenic immune pathways, while Western-style diets may promote dysbiosis, barrier dysfunction, and systemic inflammation [10,12,61]. Nutrition should therefore be considered an active determinant of disease-associated microbial patterns rather than a background lifestyle variable.

Probiotics, prebiotics, synbiotics, and postbiotics are being investigated as tools to influence this axis. Current clinical evidence is uneven and disease-specific, but these interventions may eventually serve as adjuncts that improve microbial resilience and modulate immune responses [11,61]. Postbiotics are particularly attractive because they may deliver defined metabolites or bioactive microbial products in a more controlled manner than live microbial preparations [3,61].

COVID-19 is included in this review because it represents a clinically important example of an acute infectious and inflammatory insult that can affect epithelial barriers, antiviral immune pathways, autoantibody production, and bacterial, fungal, and viral gut communities. SARS-CoV-2 infection has been associated with lasting changes in bacterial, fungal, and viral gut communities, and these changes may contribute to prolonged immune activation or to the emergence and exacerbation of autoimmune phenomena [19,22,23,64,65,66]. However, the available evidence does not establish COVID-19 as a general causal driver of autoimmune disease. Instead, COVID-19 should be viewed as a stress test of the microbiome–barrier–immune axis and as a useful model for studying how infection-associated dysbiosis may interact with genetic susceptibility and pre-existing immune instability [19,22,23].

Clinically, the diet–microbiome–immune axis should be viewed as an adjunctive and preventive framework, not as a substitute for established immunomodulatory treatment. Diet, prebiotics, probiotics, synbiotics, and postbiotics may help improve microbial resilience, barrier function, and inflammatory tone. Their use should be individualized according to disease phenotype, gastrointestinal involvement, baseline diet, medication exposure, and the patient’s microbial and metabolic profile.

## 5. Limitations and Future Directions

Several caveats are important. Most available studies are cross-sectional or observational, which limits conclusions about whether microbiome alterations cause, follow, amplify, or merely accompany autoimmune inflammation. Microbial profiles are also shaped by diet, geography, age, disease activity, antibiotics, proton pump inhibitors, corticosteroids, immunosuppressants, biologics, and non-steroidal anti-inflammatory drugs. Because these variables are not consistently controlled, they likely contribute to the inconsistent results observed across cohorts. In the present review, we therefore avoid treating disease-associated dysbiosis as direct evidence of causality unless supported by longitudinal, interventional, experimental, or mechanistic data.

Technical variation is another major source of uncertainty. Results can differ according to sample type, storage, DNA extraction method, sequencing platform, taxonomic resolution, bioinformatic pipeline, and statistical approach. The evidence base is also uneven across microbial kingdoms: bacteriome studies are substantially more numerous than mycobiome, virome, and phageome studies, and cross-kingdom interactions are rarely measured directly. Future studies should therefore use longitudinal designs, standardized dietary assessment, detailed medication recording, integrated metagenomics, metabolomics, mycobiome and virome profiling, phage-aware analyses, and parallel immune phenotyping.

The field now needs well-designed interventional trials to test whether diet- or microbiome-based strategies can modify immune activity, disease course, treatment response, or patient-reported outcomes in autoimmune disease. Such trials should distinguish clinical benefit from microbiome change alone, should measure relevant metabolites such as SCFAs, bile acids, and tryptophan derivatives, and should include safety assessment, particularly in immunosuppressed patients.

## 6. Conclusions

In summary, the gut microbiome is a biologically active interface between environmental exposure and host immune regulation in autoimmune disease. Disease-associated microbial patterns, shaped partly by nutrition, are linked to barrier dysfunction, altered metabolite signaling, molecular mimicry, and cross-kingdom interactions. The strongest evidence currently concerns bacteriome-associated mechanisms, whereas mycobiome, virome, and phageome findings remain emerging and disease dependent. Recognizing shared microbiome-associated pathways across autoimmune conditions encourages a more integrated view of pathogenesis and supports the diet–microbiome–immune axis as a plausible adjunctive therapeutic target. Nutritional modulation and microbiome-directed interventions, including probiotics, prebiotics, synbiotics, and postbiotics, may help restore microbial resilience and immune balance, but their use requires stronger mechanistic and clinical evidence. Future strategies should be personalized according to disease phenotype, gastrointestinal involvement, dietary background, medication exposure, individual microbiome signatures, and measurable metabolic readouts.

## Figures and Tables

**Table 1:** Integrated microbiome–metabolite–immune mechanisms across autoimmune diseases.

Disease	Microbiome and Metabolite Alterations	Core Immunopathogenic Mechanisms
Rheumatoid arthritis	↑ *Prevotella copri*, altered enterotype structure, ↓ beneficial commensals [30,31,32]; ↓ SCFAs and altered immune-metabolic signaling [33,34,35].	Th17/Treg imbalance, molecular mimicry, systemic inflammation [13,35,36,37].
Spondyloarthritis	Altered gut composition, ↑ pathobionts, dysbiosis linked to gut inflammation [38,39,40,41,42,43,44,45]; inflammatory microbial shifts and disturbed SCFA-related ecology [39,40,41].	Gut–joint axis activation, innate immune signaling, and subclinical intestinal inflammation [38,41].
Systemic lupus erythematosus	↑ *Ruminococcus gnavus*, altered diversity and compositional shifts [46,47,48,49]; altered bile acid- and kynurenine-related microbial signaling [49,50].	Barrier dysfunction, cytokine dysregulation, enhanced immune activation [8,12,49].
Sjögren’s syndrome	Reduced diversity and gut/oral dysbiosis [51,52,53,54]; altered mucosal microbial environment and oral–gut ecosystem changes [51,52,53,54,55,56].	Mucosal immune dysregulation, epithelial dysfunction, and glandular immune activation [53,55,56].
Systemic sclerosis	↓ *Faecalibacterium*, altered community structure, dysbiosis linked to GI involvement [57,58,59,60,61,62]; ↓ butyrate and altered microbial metabolite profiles [61,62].	Chronic inflammation, fibrosis-associated immune activation, and GI barrier dysfunction [61,62,63].
Idiopathic inflammatory myopathies	Reduced beneficial taxa and dysbiosis across bacterial, fungal, and viral communities [64,65,66,67,68,69,70]; altered immune-metabolic signatures [65,67,70].	Immune activation, muscle inflammation, and altered host–microbe signaling [65,67,70].
Systemic vasculitides	Dysbiosis described in Behçet’s disease, IgA vasculitis, Kawasaki disease, and ANCA-associated vasculitis [71,72,73,74]; ↓ butyrate-producing bacteria, altered SCFA and tryptophan metabolism [71,74].	Mucosal immune activation, IgA dysregulation, Th17/Treg imbalance, and endothelial inflammation [71,72,73,74].

↑ Increase; ↓ decrease.

**Table 2:** Nutritional and microbiome-targeted intervention strategies and translational relevance.

Disease	Nutritional and Microbiome-Targeted Interventions	Translational Relevance/Evidence
Rheumatoid arthritis	High-fiber diet, probiotics, and Mediterranean-style dietary modulation [10,11,35].	*Prevotella*-associated risk signatures and metabolite-responsive inflammation may support biomarker development and adjunctive therapeutic strategies; human + experimental evidence.
Spondyloarthritis	Diet-based microbiome modulation and probiotic/prebiotic support [10,11].	Gut-associated signatures may help refine translational targeting in SpA; human + experimental evidence.
Systemic lupus erythematosus	Polyphenol-rich and fiber-rich dietary modulation; prebiotic/probiotic approaches [10,11,34].	Microbiome composition correlates with disease activity and may provide metabolite-linked therapeutic targets; human + translational evidence.
Sjögren’s syndrome	Synbiotics and dietary strategies targeting mucosal and oral–gut microbial homeostasis [11].	Oral–gut microbiome interactions may represent a disease-relevant and clinically targetable axis; emerging clinical evidence.
Systemic sclerosis	Personalized nutrition, selective probiotic/prebiotic support, and symptom-adapted microbiome modulation [10,11].	Microbiome-based stratification may help address GI burden and fibrosis-associated inflammatory activity; limited clinical evidence.
Idiopathic inflammatory myopathies	Anti-inflammatory dietary approaches and exploratory microbiome-targeted interventions [10,11].	Early stage biomarker and mechanistic opportunities, but evidence remains preliminary.
Systemic vasculitides	Fiber-rich and plant-based diets may support SCFA production; probiotics, prebiotics, and postbiotics remain exploratory [71,72].	Gut–vascular and oral–gut axes represent emerging targets for biomarkers and adjunctive interventions [73,74]; emerging human + experimental evidence.

## Data Availability

No new data were created or analyzed in this study.

## Abbreviations

ACPA, anti-citrullinated protein antibodies; AAV, ANCA-associated vasculitis; ACR, American College of Rheumatology; ADs, autoimmune diseases; AhR, aryl hydrocarbon receptor; ANA, antinuclear antibody; ANCA, anti-neutrophil cytoplasmic antibody; AS, ankylosing spondylitis; ASAS, Assessment of SpondyloArthritis International Society; ASCA, anti-Saccharomyces cerevisiae antibodies; bEVs, bacterial extracellular vesicles; COVID-19, coronavirus disease 2019; DM, dermatomyositis; DMARD, disease-modifying antirheumatic drug; EULAR, European Alliance of Associations for Rheumatology; FODMAP, fermentable oligosaccharides, disaccharides, monosaccharides, and polyols; GI, gastrointestinal; GLP-1, glucagon-like peptide-1; HLA, human leukocyte antigen; IBD, inflammatory bowel disease; IFN, interferon; IgA, immunoglobulin A; IgG, immunoglobulin G; IL, interleukin; ILD, interstitial lung disease; IMIDs, immune-mediated inflammatory diseases; LPS, lipopolysaccharide; MCTD, mixed connective tissue disease; MD, Mediterranean diet; MTX, methotrexate; NETosis, neutrophil extracellular trap formation; NF-κB, nuclear factor kappa B; NSAIDs, non-steroidal anti-inflammatory drugs; PAH, pulmonary arterial hypertension; PAMPs, pathogen-associated molecular patterns; PBC, primary biliary cholangitis; PM, polymyositis; pSS, primary Sjögren’s syndrome; RA, rheumatoid arthritis; RF, rheumatoid factor; SARS-CoV-2, severe acute respiratory syndrome coronavirus 2; SCFAs, short-chain fatty acids; SIBO, small intestinal bacterial overgrowth; SLE, systemic lupus erythematosus; SpA, spondyloarthritis; SSc, systemic sclerosis; TGF-β, transforming growth factor beta; Th17, T helper 17 cells; TLR, Toll-like receptor; TNF-α, tumor necrosis factor alpha; Treg, regulatory T cells; VEDOSS, very early diagnosis of systemic sclerosis; 16S rRNA, 16S ribosomal RNA.

## References

[1] Deane K.D., Demoruelle M.K., Kelmenson L.B., Kuhn K.A., Norris J.M., Holers V.M. (2017). Genetic and environmental risk factors for rheumatoid arthritis. Best Pract. Res. Clin. Rheumatol..

[2] He Y., Mohapatra G., Asokan S., Nobs S.P., Elinav E. (2024). Microbiome modulation of antigen presentation in tolerance and inflammation. Curr. Opin. Immunol..

[3] Campbell C., Kandalgaonkar M.R., Golonka R.M., Yeoh B.S., Vijay-Kumar M., Saha P. (2023). Crosstalk between Gut Microbiota and Host Immunity: Impact on Inflammation and Immunotherapy. Biomedicines.

[4] Wang Y., Wei J., Zhang W., Doherty M., Zhang Y., Xie H., Li W., Wang N., Lei G., Zeng C. (2022). Gut dysbiosis in rheumatic diseases: A systematic review and meta-analysis of 92 observational studies. eBioMedicine.

[5] Tezuka H., Ohteki T. (2019). Regulation of IgA Production by Intestinal Dendritic Cells and Related Cells. Front. Immunol..

[6] Yamane S., Inagaki N. (2018). Regulation of glucagon-like peptide-1 sensitivity by gut microbiota dysbiosis. J. Diabetes Investig..

[7] Zeng Y., Wu Y., Zhang Q., Xiao X. (2024). Crosstalk between glucagon-like peptide 1 and gut microbiota in metabolic diseases. mBio.

[8] Mu Q., Kirby J., Reilly C.M., Luo X.M. (2017). Leaky Gut As a Danger Signal for Autoimmune Diseases. Front. Immunol..

[9] Marino M., Mignozzi S., Michels K.B., Cintolo M., Penagini R., Gargari G., Ciafardini C., Ferraroni M., Patel L., Del Bo’ C. (2024). Serum zonulin and colorectal cancer risk. Sci. Rep..

[10] Jiang T., Gao X., Wu C., Tian F., Lei Q., Bi J., Xie B., Wang H.Y., Chen S., Wang X. (2016). Apple-Derived Pectin Modulates Gut Microbiota, Improves Gut Barrier Function, and Attenuates Metabolic Endotoxemia in Rats with Diet-Induced Obesity. Nutrients.

[11] Ramezani Ahmadi A., Sadeghian M., Alipour M., Ahmadi Taheri S., Rahmani S., Abbasnezhad A. (2020). The Effects of Probiotic/Synbiotic on Serum Level of Zonulin as a Biomarker of Intestinal Permeability: A Systematic Review and Meta-Analysis. Iran. J. Public Health.

[12] Zhang C., Teng X., Cao Q., Deng Y., Yang M., Wang L., Rui D., Ling X., Wei C., Chen Y. (2025). Gut microbiota dysbiosis exacerbates heart failure by the LPS-TLR4/NF-κB signalling axis: Mechanistic insights and therapeutic potential of TLR4 inhibition. J. Transl. Med..

[13] Campisi L., Barbet G., Ding Y., Esplugues E., Flavell R.A., Blander J.M. (2016). Apoptosis in response to microbial infection induces autoreactive TH17 cells. Nat. Immunol..

[14] Peregrino E.S., Castañeda-Casimiro J., Vázquez-Flores L., Estrada-Parra S., Wong-Baeza C., Serafín-López J., Wong-Baeza I. (2024). The Role of Bacterial Extracellular Vesicles in the Immune Response to Pathogens, and Therapeutic Opportunities. Int. J. Mol. Sci..

[15] Lu J., Wang Y., Wu J., Duan Y., Zhang H., Du H. (2025). Linking microbial communities to rheumatoid arthritis: Focus on gut, oral microbiome and their extracellular vesicles. Front. Immunol..

[16] Lai S., Yan Y., Pu Y., Lin S., Qiu J.G., Jiang B.H., Keller M.I., Wang M., Bork P., Chen W.H. (2023). Enterotypes of the human gut mycobiome. Microbiome.

[17] Bacher P., Hohnstein T., Beerbaum E., Röcker M., Blango M.G., Kaufmann S., Röhmel J., Eschenhagen P., Grehn C., Seidel K. (2019). Human Anti-fungal Th17 Immunity and Pathology Rely on Cross-Reactivity against *Candida albicans*. Cell.

[18] Lang S., Duan Y., Liu J., Torralba M.G., Kuelbs C., Ventura-Cots M., Abraldes J.G., Bosques-Padilla F., Verna E.C., Brown R.S. (2020). Intestinal Fungal Dysbiosis and Systemic Immune Response to Fungi in Patients With Alcoholic Hepatitis. Hepatology.

[19] Cao Z., Sugimura N., Burgermeister E., Ebert M.P., Zuo T., Lan P. (2022). The gut virome: A new microbiome component in health and disease. eBioMedicine.

[20] Rybicka I., Kaźmierczak Z. (2025). The human phageome: Niche-specific distribution of bacteriophages and their clinical implications. Appl. Environ. Microbiol..

[21] Tomofuji Y., Kishikawa T., Maeda Y., Ogawa K., Nii T., Okuno T., Oguro-Igashira E., Kinoshita M., Yamamoto K., Sonehara K. (2022). Whole gut virome analysis of 476 Japanese revealed a link between phage and autoimmune disease. Ann. Rheum. Dis..

[22] Zuo T., Zhan H., Zhang F., Liu Q., Tso E.Y.K., Lui G.C.Y., Chen N., Li A., Lu W., Chan F.K.L. (2020). Alterations in Fecal Fungal Microbiome of Patients With COVID-19 During Time of Hospitalization until Discharge. Gastroenterology.

[23] Zuo T., Liu Q., Zhang F., Lui G.C., Tso E.Y., Yeoh Y.K., Chen Z., Boon S.S., Chan F.K., Chan P.K. (2021). Depicting SARS-CoV-2 faecal viral activity in association with gut microbiota composition in patients with COVID-19. Gut.

[24] Benus R.F., van der Werf T.S., Welling G.W., Judd P.A., Taylor M.A., Harmsen H.J., Whelan K. (2010). Association between *Faecalibacterium prausnitzii* and dietary fibre in colonic fermentation in healthy human subjects. Br. J. Nutr..

[25] Zhou Y., Xu H., Xu J., Guo X., Zhao H., Chen Y., Zhou Y., Nie Y.F. (2021). prausnitzii and its supernatant increase SCFAs-producing bacteria to restore gut dysbiosis in TNBS-induced colitis. AMB Express.

[26] Miquel S., Leclerc M., Martin R., Chain F., Lenoir M., Raguideau S., Hudault S., Bridonneau C., Northen T., Bowen B. (2015). Identification of metabolic signatures linked to anti-inflammatory effects of *Faecalibacterium prausnitzii*. mBio.

[27] Tan D.S.Y., Akelew Y., Snelson M., Nguyen J., O’Sullivan K.M. (2024). Unravelling the Link between the Gut Microbiome and Autoimmune Kidney Diseases: A Potential New Therapeutic Approach. Int. J. Mol. Sci..

[28] Siracusa F., Machicote A., Huber S., Gagliani N. (2026). Diet-derived microbial metabolites as regulators of immune function. Curr. Opin. Immunol..

[29] Malesza I.J., Malesza M., Walkowiak J., Mussin N., Walkowiak D., Aringazina R., Bartkowiak-Wieczorek J., Mądry E. (2021). High-Fat, Western-Style Diet, Systemic Inflammation, and Gut Microbiota: A Narrative Review. Cells.

[30] Pianta A., Arvikar S., Strle K., Drouin E.E., Wang Q., Costello C.E., Steere A.C. (2017). Evidence of the Immune Relevance of *Prevotella* copri, a Gut Microbe, in Patients With Rheumatoid Arthritis. Arthritis Rheumatol..

[31] Seifert J.A., Bemis E.A., Ramsden K., Lowell C., Polinski K., Feser M., Fleischer C., Demoruelle M.K., Buckner J., Gregersen P.K. (2023). Association of Antibodies to *Prevotella* copri in Anti-Cyclic Citrullinated Peptide-Positive Individuals At Risk of Developing Rheumatoid Arthritis and in Patients With Early or Established Rheumatoid Arthritis. Arthritis Rheumatol..

[32] Qiao J., Zhang S.-X., Chang M.-J., Cheng T., Zhang J.-Q., Zhao R., Song S., Liu G.-Y., Chang J.-S., Li X.-F. (2022). Specific enterotype of gut microbiota predicted clinical effect of methotrexate in patients with rheumatoid arthritis. Rheumatology.

[33] Zhou B., Dong C., Zhao B., Lin K., Tian Y., Zhang R., Zhu L., Xu H., Yang L. (2022). *Bacteroides* fragilis participates in the therapeutic effect of methotrexate on arthritis through metabolite regulation. Front. Microbiol..

[34] Abakar M.A.A., Awadab M.I., Elaraki Z.K.I., Osman M.E., Ibrahim-Holi M.A., Ahmad-Abakur E.H., Alnour T.M.S. (2025). Rheumatoid Factor and Anti-CCP Titers in COVID-19. Infect. Disord. Drug Targets.

[35] Pang A., Pu S., Pan Y., Huang N., Li D. (2025). Short-chain fatty acids from gut microbiota restore Th17/Treg balance in rheumatoid arthritis: Mechanisms and therapeutic potential. J. Transl. Autoimmun..

[36] Sherina N., de Vries C., Kharlamova N., Sippl N., Jiang X., Brynedal B., Kindstedt E., Hansson M., Mathsson-Alm L., Israelsson L. (2022). Antibodies to a Citrullinated Porphyromonas gingivalis Epitope Are Increased in Early Rheumatoid Arthritis, and Can Be Produced by Gingival Tissue B Cells: Implications for a Bacterial Origin in RA Etiology. Front. Immunol..

[37] Muruganandam A., Migliorini F., Jeyaraman N., Vaishya R., Balaji S., Ramasubramanian S., Maffulli N., Jeyaraman M. (2024). Molecular Mimicry Between Gut Microbiome and Rheumatoid Arthritis: Current Concepts. Med. Sci..

[38] Gracey E., Vereecke L., McGovern D., Fröhling M., Schett G., Danese S., De Vos M., Van den Bosch F., Elewaut D. (2020). Revisiting the gut-joint axis: Links between gut inflammation and spondyloarthritis. Nat. Rev. Rheumatol..

[39] Marquez-Ortiz R.A., Leon M., Abril D., Escobar-Perez J., Florez-Sarmiento C., Parra-Izquierdo V., Chalem P., Romero-Sanchez C. (2023). Colonoscopy aspiration lavages for mucosal metataxonomic profiling of spondylarthritis-associated gastrointestinal tract alterations. Sci. Rep..

[40] Klingberg E., Magnusson M.K., Strid H., Deminger A., Ståhl A., Sundin J., Simrén M., Carlsten H., Öhman L., Forsblad-d’Elia H. (2019). A distinct gut microbiota composition in patients with ankylosing spondylitis is associated with increased levels of fecal calprotectin. Arthritis Res. Ther..

[41] Vereecke L., Elewaut D. (2017). Spondyloarthropathies: Ruminococcus on the horizon in arthritic disease. Nat. Rev. Rheumatol..

[42] Wang X., Sun J., Zhang X., Chen W., Cao J., Hu H. (2024). Metagenomics reveals unique gut mycobiome biomarkers in psoriasis. Ski. Res. Technol..

[43] Li X.V., Leonardi I., Putzel G.G., Semon A., Fiers W.D., Kusakabe T., Lin W.-Y., Gao I.H., Doron I., Gutierrez-Guerrero A. (2022). Immune regulation by fungal strain diversity in inflammatory bowel disease. Nature.

[44] Li M., Dai B., Tang Y., Lei L., Li N., Liu C., Ge T., Zhang L., Xu Y., Hu Y. (2019). Altered Bacterial-Fungal Interkingdom Networks in the Guts of Ankylosing Spondylitis Patients. mSystems.

[45] Li C., Zhang Y., Yan Q., Guo R., Chen C., Li S., Zhang Y., Meng J., Ma J., You W. (2023). Alterations in the gut virome in patients with ankylosing spondylitis. Front. Immunol..

[46] Azzouz D., Omarbekova A., Heguy A., Schwudke D., Gisch N., Rovin B.H., Caricchio R., Buyon J.P., Alekseyenko A.V., Silverman G.J. (2019). Lupus nephritis is linked to disease-activity associated expansions and immunity to a gut commensal. Ann. Rheum. Dis..

[47] Zhu J.H., Wu L.P., Deng L., Zang S.G., Li X.B., Chen X., Yu J.X. (2025). Gut microbiota and metabolism in systemic lupus erythematosus: From dysbiosis to targeted interventions. Eur. J. Med. Res..

[48] Medina-Martínez I., Gil-Gutiérrez R., García-García J., de la Hera-Fernández F.J., Navarrete-Navarrete N., Zamora-Pasadas M., Ortego-Centeno N., Callejas-Rubio J.L., García-García F., Gálvez-Peralta J. (2025). Association of Gut Dysbiosis with Disease Phenotype and Treatment in Systemic Lupus Erythematosus. Med. Sci..

[49] Garcia A.C., Six N., Ma L., Morel L. (2024). Intersection of the microbiome and immune metabolism in lupus. Immunol. Rev..

[50] Wang Z., Xing Y., Xu M., Chen C., Zhu Q., Chen H., Zhang Y., Chen W., Feng J., Zhang A. (2025). Altered gut mycobiome and cross-kingdom microbial interactions in systemic lupus erythematosus. J. Transl. Med..

[51] Mandl T., Marsal J., Olsson P., Ohlsson B., Andréasson K. (2017). Severe intestinal dysbiosis is prevalent in primary Sjögren’s syndrome and is associated with systemic disease activity. Arthritis Res. Ther..

[52] Zang B., Xu L., Huang H., Liu Q., Yao Y., Li J., Yang Y., Zhao C., Liu B., Liu B. (2025). Decoding the Gut Microbiome in Primary Sjögren’s Syndrome and Primary Biliary Cholangitis: Shared Dysbiosis, Distinct Patterns, and Associations with Clinical Features. Microorganisms.

[53] Tseng Y.-C., Yang H.-Y., Lin W.-T., Chang C.-B., Chien H.-C., Wang H.-P., Chen C.-M., Wang J.-T., Li C., Wu S.-F. (2021). Salivary dysbiosis in Sjögren’s syndrome and a commensal-mediated immunomodulatory effect of salivary gland epithelial cells. npj Biofilms Microbiomes.

[54] Saúco C., Rus M.J., Nieto M.R., Barros C., Cantiga-Silva C., Lendines-Cordero D., Calderer-Ortiz M., Zurita-García M., Arias-Herrera S., Monsalve-Guil L. (2023). Hyposalivation but not Sjögren’s syndrome associated with microbial dysbiosis in women. Front. Microbiol..

[55] Chen C.L., Chang F.C., Hung Y.M., Chou M.C., Yip H.T., Chang R., Wei J.C. (2021). *Candida* Infection as an Early Sign of Subsequent Sjögren’s Syndrome: A Population-Based Matched Cohort Study. Front. Med..

[56] Zhang X., Li Y., Qin Y., Deng C., Shi G., Liu Y. (2025). OP0148 Salivary Virome Alterations In Sjögren’s Disease Contribute to Immune Homeostasis Disruption by Molecular Mimicry. Ann. Rheum. Dis..

[57] Sattar B., Chokshi R.V. (2019). Colonic and Anorectal Manifestations of Systemic Sclerosis. Curr. Gastroenterol. Rep..

[58] Patrone V., Puglisi E., Cardinali M., Schnitzler T.S., Svegliati S., Festa A., Gabrielli A., Morelli L. (2017). Gut microbiota profile in systemic sclerosis patients with and without clinical evidence of gastrointestinal involvement. Sci. Rep..

[59] Natalello G., Bosello S.L., Paroni Sterbini F., Posteraro B., De Lorenzis E., Canestrari G.B., Gigante L., Verardi L., Ferraccioli G., Sanguinetti M. (2020). Gut microbiota analysis in systemic sclerosis according to disease characteristics and nutritional status. Clin. Exp. Rheumatol..

[60] Levin D., De Palma G., Zou H., Bazzaz A.H.Z., Verdu E., Baker B., Pinto-Sanchez M.I., Khalidi N., Larché M.J., Beattie K.A. (2021). Fecal microbiome differs between patients with systemic sclerosis with and without small intestinal bacterial overgrowth. J. Scleroderma Relat. Disord..

[61] Bellando-Randone S., Russo E., Di Gloria L., Lepri G., Baldi S., Fioretto B.S., Romano E., Ghezzi G., Bertorello S., El Aoufy K. (2024). Gut microbiota in very early systemic sclerosis: The first case-control taxonomic and functional characterisation highlighting an altered butyric acid profile. RMD Open.

[62] Andréasson K., Lee S.M., Lagishetty V., Wu M., Howlett N., English J., Hesselstrand R., Clements P.J., Jacobs J.P., Volkmann E.R. (2022). Disease Features and Gastrointestinal Microbial Composition in Patients with Systemic Sclerosis from Two Independent Cohorts. ACR Open Rheumatol..

[63] Kim S., Park H.J., Lee S.I. (2022). The Microbiome in Systemic Sclerosis: Pathophysiology and Therapeutic Potential. Int. J. Mol. Sci..

[64] Zhufeng Y., Xu J., Miao M., Wang Y., Li Y., Huang B., Guo Y., Tian J., Sun X., Li J. (2022). Modification of Intestinal Microbiota Dysbiosis by Low-Dose Interleukin-2 in Dermatomyositis: A Post Hoc Analysis From a Clinical Trial Study. Front. Cell. Infect. Microbiol..

[65] Niu Y., Zhang Y., Fan K., Hou J., Liu L., Zhang H., Geng X., Ma X., Lin S., Guo M. (2024). Genetically predicted the causal relationship between gut microbiota and the risk of polymyositis/dermatomyositis: A Mendelian randomization analysis. Front. Microbiol..

[66] Winkler M., Seel W., Kornblum C., Simon M.C., Reimann J. (2026). The MicroIBioM study: The gut microbiome in inclusion body myositis. Clin. Exp. Rheumatol..

[67] Bae S.S., Dong T.S., Wang J., Lagishetty V., Katzka W., Jacobs J.P., Charles-Schoeman C. (2022). Altered Gut Microbiome in Patients With Dermatomyositis. ACR Open Rheumatol..

[68] Koester S.T., Chow A., Pepper-Tunick E., Lee P., Eckert M., Brenchley L., Gardner P., Song H.J., Li N., Schiffenbauer A. (2024). Familial clustering of dysbiotic oral and fecal microbiomes in juvenile dermatomyositis. Sci. Rep..

[69] Massa M., Costouros N., Mazzoli F., De Benedetti F., La Cava A., Le T., De Kleer I., Ravelli A., Liotta M., Roord S. (2002). Self epitopes shared between human skeletal myosin and *Streptococcus* pyogenes M5 protein are targets of immune responses in active juvenile dermatomyositis. Arthritis Rheum..

[70] Liu C., Xing Y., Su J., Liu Y., Dou Y., Wang Z., Sha S., Yan Q., Xu M., Zhao L. (2026). Multi-kingdom gut microbiota characterization in Chinese patients with idiopathic inflammatory myopathies. Sci. Rep..

[71] Chen H., Liu H., Qiao L., Liu Y., Yu D., Wang Z., Wang T., Li W. (2025). The characteristics of the gut microbiota in patients with Kawasaki disease: A systematic review. Front. Microbiol..

[72] Joubert M., André M., Barnich N., Billard E. (2023). Microbiome and Behçet’s disease: A systematic review. Clin. Exp. Rheumatol..

[73] Sun B., He X., Zhang W. (2022). Findings on the Relationship Between Intestinal Microbiome and Vasculitis. Front. Cell. Infect. Microbiol..

[74] Hu X., Fan R., Song W., Qing J., Yan X., Li Y., Duan Q., Li Y. (2022). Landscape of intestinal microbiota in patients with IgA nephropathy, IgA vasculitis and Kawasaki disease. Front. Cell. Infect. Microbiol..

[75] Gravallese E.M., Firestein G.S. (2023). Rheumatoid Arthritis—Common Origins, Divergent Mechanisms. N. Engl. J. Med..

[76] Bonfiglioli K.R., de Medeiros Ribeiro A.C., Carnieletto A.P., Pereira I., Domiciano D.S., da Silva H.C., Pugliesi A., Pereira L.R., Guimarães M.F.R., Giorgi R.D.N. (2023). Extra-articular manifestations of rheumatoid arthritis remain a major challenge: Data from a large, multi-centric cohort. Adv. Rheumatol..

[77] Aletaha D., Neogi T., Silman A.J., Funovits J., Felson D.T., Bingham C.O., Birnbaum N.S., Burmester G.R., Bykerk V.P., Cohen M.D. (2010). 2010 Rheumatoid arthritis classification criteria: An American College of Rheumatology/European League Against Rheumatism collaborative initiative. Ann. Rheum. Dis..

[78] Sokolova M.V., Schett G., Steffen U. (2022). Autoantibodies in Rheumatoid Arthritis: Historical Background and Novel Findings. Clin. Rev. Allergy Immunol..

[79] McInnes I.B., Gravallese E.M. (2021). Immune-mediated inflammatory disease therapeutics: Past, present and future. Nat. Rev. Immunol..

[80] Li Y., Guo R., Oduro P.K., Sun T., Chen H., Yi Y., Zeng W., Wang Q., Leng L., Yang L. (2022). The Relationship Between Porphyromonas Gingivalis and Rheumatoid Arthritis: A Meta-Analysis. Front. Cell. Infect. Microbiol..

[81] Flak M.B., Colas R.A., Muñoz-Atienza E., Curtis M.A., Dalli J., Pitzalis C. (2019). Inflammatory arthritis disrupts gut resolution mechanisms, promoting barrier breakdown by Porphyromonas gingivalis. JCI Insight.

[82] Onuora S. (2019). *P. gingivalis* exacerbates arthritis via gut barrier dysfunction. Nat. Rev. Rheumatol..

[83] Sun X., Wang Y., Li X., Wang M., Dong J., Tang W., Lei Z., Guo Y., Li M., Li Y. (2022). Alterations of gut fungal microbiota in patients with rheumatoid arthritis. PeerJ.

[84] Guo R., Li S., Zhang Y., Zhang Y., Wang G., Ullah H., Ma Y., Yan Q. (2022). Dysbiotic Oral and Gut Viromes in Untreated and Treated Rheumatoid Arthritis Patients. Microbiol. Spectr..

[85] Hu F., Li X., Liu K., Li Y., Xie Y., Wei C., Liu S., Song J., Wang P., Shi L. (2024). Rheumatoid arthritis patients harbour aberrant enteric bacteriophages with autoimmunity-provoking potential: A paired sibling study. Ann. Rheum. Dis..

[86] Mangalea M.R., Paez-Espino D., Kieft K., Chatterjee A., Chriswell M.E., Seifert J.A., Feser M.L., Demoruelle M.K., Sakatos A., Anantharaman K. (2021). Individuals at risk for rheumatoid arthritis harbor differential intestinal bacteriophage communities with distinct metabolic potential. Cell Host Microbe.

[87] Marín J.S., Mazenett-Granados E.A., Salazar-Uribe J.C., Sarmiento M., Suárez J.F., Rojas M., Munera M., Pérez R., Morales C., Dominguez J.I. (2023). Increased incidence of rheumatoid arthritis after COVID-19. Autoimmun. Rev..

[88] Bouden S., Ben Ayed H., Rouached L., Ben Tekaya A., Mahmoud I., Tekaya R., Saidane O., Abdelmoula L. (2024). COVID-19-Induced Rheumatoid Arthritis: Case Series and Systematic Review of the Literature. Mediterr. J. Rheumatol..

[89] Lamacchia C., Gilbert B., Studer O., Lauper K., Finckh A. (2023). Brief report: Can COVID-19 infection trigger rheumatoid arthritis-associated autoimmunity in individuals at risk for the disease? A nested cohort study. Front. Med..

[90] Rudwaleit M., van der Heijde D., Landewé R., Listing J., Akkoc N., Brandt J., Braun J., Chou C.T., Collantes-Estevez E., Dougados M. (2009). The development of Assessment of SpondyloArthritis international Society classification criteria for axial spondyloarthritis (part II): Validation and final selection. Ann. Rheum. Dis..

[91] van der Linden S., Valkenburg H.A., Cats A. (1984). Evaluation of diagnostic criteria for ankylosing spondylitis. A proposal for modification of the New York criteria. Arthritis Rheum..

[92] Rudwaleit M., van der Heijde D., Landewé R., Akkoc N., Brandt J., Chou C.T., Dougados M., Huang F., Gu J., Kirazli Y. (2011). The Assessment of SpondyloArthritis international Society classification criteria for peripheral spondyloarthritis and for spondyloarthritis in general. Ann. Rheum. Dis..

[93] Gopalarathinam R., Nawimana S., Nune A. (2023). Axial spondylarthritis following COVID-19 infection. BMJ Case Rep..

[94] Oh M., Kim J.G., Baek I.-P., Ju J.H. (2023). Development of Spondyloarthritis After COVID-19 in HLAB27-Positive Monozygotic Twins: Case Reports With Single Cell Transcriptome Profiling. J. Rheum. Dis..

[95] Aringer M., Costenbader K., Daikh D., Brinks R., Mosca M., Ramsey-Goldman R., Smolen J.S., Wofsy D., Boumpas D.T., Kamen D.L. (2019). 2019 European League Against Rheumatism/American College of Rheumatology Classification Criteria for Systemic Lupus Erythematosus. Arthritis Rheumatol..

[96] Christovich A., Luo X.M. (2022). Gut Microbiota, Leaky Gut, and Autoimmune Diseases. Front. Immunol..

[97] Cheibchalard T., Leelahavanichkul A., Chatthanathon P., Klankeo P., Hirankarn N., Somboonna N. (2024). Fungal microbiome in gut of systemic lupus erythematosus (SLE)-prone mice (pristane and FCGRIIb deficiency), a possible impact of fungi in lupus. PLoS ONE.

[98] Chen B., Cao J., Liu W., Zhang Y., Liu Y., Wang M., Xiao F., Ma J., Wang J., Zhang X. (2023). Disturbed gut virome with potent interferonogenic property in systemic lupus erythematosus. Sci. Bull..

[99] Ramachandran L., Dontaraju V.S., Troyer J., Sahota J. (2022). New onset systemic lupus erythematosus after COVID-19 infection: A case report. AME Case Rep..

[100] Hejazian S.S., Hejazian S.M., Farnood F., Abedi Azar S. (2022). Dysregulation of immunity in COVID-19 and SLE. Inflammopharmacology.

[101] Shiboski C.H., Shiboski S.C., Seror R., Criswell L.A., Labetoulle M., Lietman T.M., Rasmussen A., Scofield H., Vitali C., Bowman S.J. (2017). 2016 American College of Rheumatology/European League Against Rheumatism Classification Criteria for Primary Sjögren’s Syndrome: A Consensus and Data-Driven Methodology Involving Three International Patient Cohorts. Arthritis Rheumatol..

[102] Bartoloni E., Bistoni O., Alunno A., Cavagna L., Nalotto L., Baldini C., Priori R., Fischetti C., Fredi M., Quartuccio L. (2019). Celiac Disease Prevalence Is Increased in Primary Sjögren’s Syndrome and Diffuse Systemic Sclerosis: Lessons from a Large Multi-Center Study. J. Clin. Med..

[103] Bakhsh R., Dairi K., Almadabgy E., Albiladi A., Gamal L., Almatrafi D., AlShariff F., Alsefri A. (2024). New Onset of Neuro-Sjögren’s Syndrome Nine Months After the Third COVID-19 Vaccine Dose: A Case Report. Cureus.

[104] Cañadillas Sánchez E., Oliver García E.M., del Río Martínez P.S. (2024). Overlap of sarcoidosis and Sjögren’s syndrome triggered by COVID-19 infection. Med. Clín. (Engl. Ed.).

[105] Shen Y., Voigt A., Goranova L., Abed M., Kleiner D.E., Maldonado J.O., Beach M., Pelayo E., Chiorini J.A., Craft W.F. (2023). Evidence of a Sjögren’s disease–like phenotype following COVID-19 in mice and humans. JCI Insight.

[106] Felix F.A., Jiang Y., Zhou J., Li D., Zhou V., de Sousa S.F., Byrd K.M., Yu Q. (2025). SARS-CoV-2 spike protein induces salivary gland dysfunction and immune infiltration in C57BL/6 mice. Front. Immunol..

[107] Zhu Y., Zhang K., Luo Z., Song Y., Wang X. (2025). Gut microbiota on anxiety and depression in primary Sjogren’s syndrome: A novel insight. Heliyon.

[108] Carroll M., Nagarajah V., Campbell S. (2023). Systemic sclerosis following COVID-19 infection with recurrent corticosteroid-induced scleroderma renal crisis. BMJ Case Rep..

[109] Zou H., Rau A., Daveluy S. (2023). Scleroderma after COVID-19 Infection and Vaccination. Skinmed.

[110] Matucci-Cerinic M., Hughes M., Taliani G., Kahaleh B. (2021). Similarities between COVID-19 and systemic sclerosis early vasculopathy: A “viral” challenge for future research in scleroderma. Autoimmun. Rev..

[111] Lundberg I.E., Tjärnlund A., Bottai M., Werth V.P., Pilkington C., de Visser M., Alfredsson L., Amato A.A., Barohn R.J., Liang M.H. (2017). 2017 European League Against Rheumatism/American College of Rheumatology classification criteria for adult and juvenile idiopathic inflammatory myopathies and their major subgroups. Arthritis Rheumatol..

[112] Saud A., Naveen R., Aggarwal R., Gupta L. (2021). COVID-19 and Myositis: What We Know So Far. Curr. Rheumatol. Rep..

[113] García-Bravo L., Prada A., Gutiérrez Larrañaga M., Espinosa Ros E., Almeida González D., Martín Martínez D., Rodríguez Sánchez T., Mingorance Gámez C.G., Jurado Roger A., Aguado Álvarez R. (2024). Increased Risk of Myositis-Specific and Myositis-Associated Autoantibodies After COVID-19 Pandemic and Vaccination: A Spanish Multicenter Collaborative Study. Biomedicines.

[114] Jennette J.C., Falk R.J., Bacon P.A., Basu N., Cid M.C., Ferrario F., Flores-Suarez L.F., Gross W.L., Guillevin L., Hagen E.C. (2013). 2012 revised International Chapel Hill Consensus Conference Nomenclature of Vasculitides. Arthritis Rheum..

[115] Dekkema G.J., Rutgers A., Sanders J.S., Stegeman C.A., Heeringa P. (2021). The Nasal Microbiome in ANCA-Associated Vasculitis: Picking the Nose for Clues on Disease Pathogenesis. Curr. Rheumatol. Rep..

[116] Najem C., Lee J.-J., Tanes C., Strange C., Friedman E., Sreih A., Rhee R., Geara A., Hongzhe L., Bittinger K. (2019). SAT0623 Characterizing the Gut and Plasma Metabolomes in Patients with ANCA-Associated Vasculitis. Ann. Rheum. Dis..

[117] Yu B., Jin L., Chen Z., Nie W., Chen L., Ma Y., Chen H., Wu Y., Ma Y., Chen J. (2021). The gut microbiome in microscopic polyangiitis with kidney involvement: Common and unique alterations, clinical association and values for disease diagnosis and outcome prediction. Ann. Transl. Med..

[118] Wu M., Liao Z., Zeng K., Jiang Q. (2023). Exploring the causal role of gut microbiota in giant cell arteritis: A Mendelian randomization analysis with mediator insights. Front. Immunol..

[119] Han Z., Hu J., Zhang Z., Yu Q., He P.F., Li X., Zhang S.X. (2023). AB0171 Mendelian Randomization Analysis Reveals Causal Effects of Gut Microbiota Abundance on Giant Cell Arteritis Risk. Ann. Rheum. Dis..

[120] Fan L., Chen J., Pan L., Xin X., Geng B., Yang L., Wang Q., Ma W., Lou Y., Bian J. (2023). Alterations of Gut Microbiome, Metabolome, and Lipidome in Takayasu Arteritis. Arthritis Rheumatol..

[121] Barber T.M., Kabisch S., Pfeiffer A.F.H., Weickert M.O. (2023). The Effects of the Mediterranean Diet on Health and Gut Microbiota. Nutrients.

[122] Perrone P., D’Angelo S. (2025). Gut Microbiota Modulation Through Mediterranean Diet Foods: Implications for Human Health. Nutrients.

[123] Ricchi M., Odoardi M.R., Carulli L., Anzivino C., Ballestri S., Pinetti A., Fantoni L.I., Marra F., Bertolotti M., Banni S. (2009). Differential effect of oleic and palmitic acid on lipid accumulation and apoptosis in cultured hepatocytes. J. Gastroenterol. Hepatol..

[124] Di Giuseppe D., Wallin A., Bottai M., Askling J., Wolk A. (2014). Long-term intake of dietary long-chain n-3 polyunsaturated fatty acids and risk of rheumatoid arthritis: A prospective cohort study of women. Ann. Rheum. Dis..

[125] Jiang J., Li K., Wang F., Yang B., Fu Y., Zheng J., Li D. (2016). Effect of Marine-Derived n-3 Polyunsaturated Fatty Acids on Major Eicosanoids: A Systematic Review and Meta-Analysis from 18 Randomized Controlled Trials. PLoS ONE.

[126] Hu P., Lee E.K.-P., Li Q., Tam L.-S., Wong S.Y.-S., Poon P.K.-M., Yip B.H.-K. (2025). Mediterranean diet and rheumatoid arthritis: A nine-year cohort study and systematic review with meta-analysis. Eur. J. Clin. Nutr..

[127] Hu Y., Costenbader K.H., Gao X., Hu F.B., Karlson E.W., Lu B. (2015). Mediterranean Diet and Incidence of Rheumatoid Arthritis in Women. Arthritis Care Res..

[128] Raad T., George E., Griffin A., Larkin L., Fraser A., Kennedy N., Tierney A. (2024). Effects of a telehealth-delivered Mediterranean diet intervention in adults with Rheumatoid Arthritis (MEDRA): A randomised controlled trial. BMC Musculoskelet. Disord..

[129] Virvili A., Koletsos N., Gerolymatou N., Memi T.E., Karakosta M., Voulgari P.V. (2025). POS0546 Mediterranean Diet and Rheumatoid Arthritis: A Controlled Trial. Ann. Rheum. Dis..

[130] Pocovi-Gerardino G., Correa-Rodríguez M., Callejas-Rubio J.L., Ríos-Fernández R., Martín-Amada M., Cruz-Caparros M.G., Rueda-Medina B., Ortego-Centeno N. (2021). Beneficial effect of Mediterranean diet on disease activity and cardiovascular risk in systemic lupus erythematosus patients: A cross-sectional study. Rheumatology.

[131] Gavilán-Carrera B., Aguilera-Fernández V., Amaro-Gahete F.J., Rosales-Castillo A., Soriano-Maldonado A., Vargas-Hitos J.A. (2024). Association of the Mediterranean diet with arterial stiffness, inflammation, and medication use in women with systemic lupus erythematosus: An exploratory study. J. Nutr. Biochem..

[132] Merra N., Osele A.G., Hulej G., Ragno D., Bindoli S., Oliviero F., Ramonda R., Zen M. (2026). LBA:01:09 Mediterranean diet and systemic lupus erythematosus: Impact on disease severity and cardiovascular risk. Lupus Sci. Med..

[133] Nassani B.M., Eid H., El Tahech S., El Alam A., Chaaya C., Maalouly G. (2026). Mediterranean diet and symptom severity in Sjogren’s syndrome. Front. Med..

[134] Chaaya C., Raad E., Kahale F., Chelala E., Ziade N., Maalouly G. (2025). Adherence to Mediterranean Diet and Ocular Dryness Severity in Sjögren’s Syndrome: A Cross-Sectional Study. Med. Sci..

[135] Machowicz A., Hall I., de Pablo P., Rauz S., Richards A., Higham J., Poveda-Gallego A., Imamura F., Bowman S.J., Barone F. (2020). Mediterranean diet and risk of Sjögren’s syndrome. Clin. Exp. Rheumatol..

[136] Natalello G., Bosello S.L., Campochiaro C., Abignano G., De Santis M., Ferlito A., Karadağ D.T., Padula A.A., Cavalli G., D’Agostino M.A. (2024). Adherence to the Mediterranean Diet in Italian Patients With Systemic Sclerosis: An Epidemiologic Survey. ACR Open Rheumatol..

[137] Cano-Garcia L., Garcia Studer A., Ortiz-Márquez F., Manrique-Arija S., Mena-Vázquez N., Fernández-Nebro A. (2025). ABS0465 Impact of Adherence to the Mediterranean Diet on the Severity and Functionality of Patient with Systemic Sclerosis. Ann. Rheum. Dis..

[138] Ometto F., Ortolan A., Farber D., Lorenzin M., Dellamaria G., Cozzi G., Favero M., Valentini R., Doria A., Ramonda R. (2021). Mediterranean diet in axial spondyloarthritis: An observational study in an Italian monocentric cohort. Arthritis Res. Ther..

[139] Bustamante M.F., Agustín-Perez M., Cedola F., Coras R., Narasimhan R., Golshan S., Guma M. (2020). Design of an anti-inflammatory diet (ITIS diet) for patients with rheumatoid arthritis. Contemp. Clin. Trials Commun..

[140] Climent M.S., Cedeno M., Coras R., Holt T., Choi S.I., Singh A., Nguyen K.N., Lee S., Zuffa S.Z., Agustin-Perez M. (2023). Comparing the ITIS Diet and the Mediterranean Diet in Rheumatoid Arthritis: Preliminary Findings on Clinical and Microbiome Outcomes. Arthritis Rheumatol..

[141] Pardali E.C., Gkouvi A., Gkouskou K.K., Manolakis A.C., Tsigalou C., Goulis D.G., Bogdanos D.P., Grammatikopoulou M.G. (2025). Autoimmune protocol diet: A personalized elimination diet for patients with autoimmune diseases. Metab. Open.

[142] Nguyen A.D., Andréasson K., McMahan Z.H., Bukiri H., Howlett N., Lagishetty V., Lee S.M., Jacobs J.P., Volkmann E.R. (2023). Gastrointestinal tract involvement in systemic sclerosis: The roles of diet and the microbiome. Semin. Arthritis Rheum..

